# Prevalence and contributing factors of birth asphyxia among the neonates delivered at Nigist Eleni Mohammed memorial teaching hospital, Southern Ethiopia: a cross-sectional study

**DOI:** 10.1186/s12884-019-2696-6

**Published:** 2019-12-30

**Authors:** Ritbano Ahmed Abdo, Hassen Mosa Halil, Biruk Assefa Kebede, Abebe Alemu Anshebo, Negeso Gebeyehu Gejo

**Affiliations:** Department of Midwifery, College of Medicine and Health Sciences, Wachemo University, Hossana, Ethiopia

**Keywords:** Birth asphyxia, Prevalence, Contributing factors

## Abstract

**Background:**

Birth asphyxia is a major contributor to neonatal mortality worldwide. In Ethiopia, birth asphyxia remains a severe condition that leads to significant mortality and morbidity. This study aims to assess the prevalence and contributing factors of birth asphyxia among the neonates delivered at the Nigist Eleni Mohammed Memorial Teaching Hospital, Southern Ethiopia.

**Methods:**

This hospital-based cross-sectional study was carried out on 279 participants using the systematic sampling method during June 1–30, 2019. Data were collected using a pretested structured interviewer administered questionnaire, check list and chart review, which was used to retrieve medical information and mother’s test results that could not be captured by the interview**.** Data were entered into EpiData (version 3.1) and analyzed using SPSS software (version 24). Multivariable regression analysis was used to identify the association between the independent variables and outcome variable with a 95% confidence interval (CI).

**Result:**

The overall prevalence of birth asphyxia among newborns was found to be 15.1%. Factors that were significantly associated with birth asphyxia included mothers aged ≥35 (AOR = 6.4; 95% CI = 2.0–20.5), primigravida (AOR = 5.1; 95% CI =2.0–13.3), prolonged second stage of labor (AOR = 4.6; 95%CI =1.6–13.3), preterm birth (AOR = 4.7; 95% CI =1.5–14.1), meconium stained amniotic fluid (AOR = 7.5; 95% CI =2.5–21.4) and tight nuchal (AOR = 3.1; 95% CI =1.2–9.3).

**Conclusion:**

Birth asphyxia is still prevalent in the study setting. The obtained findings indicated that the mothers aged ≥35, being primigravida, preterm birth, meconium stained amniotic fluid and tight nuchal were the factors associated with birth asphyxia. The results of this study show the need for better maternal care, creating awareness about contributing factors of birth asphyxia to the maternity health professionals, careful monitoring of labor, and identifying and taking proper measures that could help in reducing the occurrence of birth asphyxia.

## Background

Birth asphyxia is defined by the World Health Organisation (WHO) as ‘the failure to initiate and sustain breathing at birth’ [1]. It is a major contributor to neonatal mortality worldwide [2–5], causing 24% of all neonatal deaths [3] and 11% of deaths of children under 5 years of age [2]. Almost all asphyxia related deaths (98%) occur during the first week of life [6]. About 75% of such deaths occur on the first day, and less than 2% after 72 h of birth [5].

Birth asphyxia is a leading cause of brain damage. If severe, it can injure brain cells and cause potentially fatal conditions, including hypoxic-ischemic encephalopathy, brain injuries, autism, attention deficit hyperactivity disorder, seizures, and cerebral palsy [7]. Survivors often face lifelong health problems (80%), such as disabilities, developmental delays, palsy, intellectual disabilities, and behavioural problems [7–10]. Furthermore, birth asphyxia places financial and emotional burdens on the families and communities involved [10, 11].

Birth asphyxia is associated with a complex range of risk factors, which vary from industrialised and non-industrialised countries. These factors are grouped according to whether they are before birth (antepartum risk actors), during birth (intrapartum risk factors), or after birth (postpartum risk factors) [12]. Antepartum risk factors include severe maternal hypotension or hypertensive diseases during pregnancy [13, 14], antepartum haemorrhage [15, 16], history of stillbirth [17], fewer than four antenatal care visits [14, 15, 17], oligohydramnios [14], maternal fever [13, 15, 18], and maternal anaemia [13, 14, 17], young maternal age [13, 18, 19], advanced maternal age [20], and low educational status (illiterate and primary education level) [17, 19]. Intrapartum risk factors include malpresentation [13–15, 17, 21], prolonged second stage of labour [15, 16, 22], home delivery [13, 18], obstructed labour [13, 18, 23], oxytocin use [16], and meconium stained amniotic fluid [13–15, 18]. Fetal risk factors associated with birth asphyxia include low birth weight [15, 18], high birth weight [22], multiple gestation [14], tight nuchal cord [15, 22], preterm delivery [13, 14, 22], resuscitation, and fetal distress [17, 24].

The Federal Ministry of Health developed the first comprehensive National Child Survival Strategy (2015–2020) in 2015, aiming to reduce under-five mortality by two thirds. Since its development in 2015, several evidence-based interventions that need to be incorporated in the strategy have been initiated. These include community management of pneumonia through integrated community case management, community-based newborn sepsis management through community-based newborn care, newborn intensive care units, newborn corners, and the introduction of Haemophilus influenza, pneumococcal, and shifting PMTCT to ‘Option B+’ [25]. The guidelines to treat birth asphyxia are also well established in Ethiopia and made available even at the health-centre level for assessing and classifying [26].

Despite this, a high number of newborn deaths due to birth asphyxia were reported in Ethiopia. From observations thus far, the issue of birth asphyxia remains unresolved. Ethiopia’s rate of neonatal mortality is still among the highest in sub-Saharan Africa [27, 28]. In 2015, nearly 240 babies in their first month died each day [29]. Birth asphyxia is the leading cause of neonatal deaths and accounts for about 31.6% of neonatal mortality, followed by preterm birth (21.8%) and sepsis (18.5%) [30]. Moreover, the findings from studies conducted in Tigray [31] and Gondor [32] to identify causes of neonatal mortality revealed that birth asphyxia was responsible for 35 and 12.5% of neonatal deaths respectively.

Analysis of birth asphyxia is important because it may reveal possible factors. Therefore, this study aims to assess the prevalence and contributing factors of birth asphyxia among neonates delivered in the Nigist Eleni Mohammed Memorial Teaching Hospital, Southern Ethiopia.

## Methods

### Study setting, population, sample seize and technique

This hospital-based cross-sectional study was undertaken at Nigist Eleni Mohammed Memorial Teaching Hospital in the Hossana Town, during June 1–30 in 2019. The single population proportion formula was used to determine the sample size with the following assumptions; the proportion of birth asphyxia was taken from the study conducted as Jimma Public Hospitals (12.5%) [15], with a 95% confidence interval (CI), margin error of 4, and 5% non- response rate. Finally, the sample size was obtained at 279 for the present study using the systematic sampling technique. Every third woman from the delivery registration book, who delivered ≥ 28 weeks with a live infant, was selected as a study subject. If a mother gave birth to more than one baby, one of these babies was selected using a simple random sampling method.

### Data collection procedure

Data were collected using a combination of a pretested structured interviewer-administered questionnaire, observation check list and client’s chart reviewed, which was used to retrieve medical information and mother’s test results that could not be captured by the interview. The questionnaire was developed based on the instrument that was applied in other related studies [15–22, 31, 32]. It was structured into four parts, namely, sociodemographic characteristics, antepartum factors, intrapartum factors and fetal condition and postpartum factors. Three BSc. degree midwives who are able to speak both Hadiyisa (local language) and Amharic were recruited for the data collection, and three MSc. midwives participated in supervising the data collectors. Data collection began from the admission of a mother to the labour ward and lasted till the 5th minute after delivery. Data were collected throughout the day (including night). Data collectors followed the maternal conditions, labour progress and fetal conditions during follow up. The fetal heart rate was monitored through fetoschope and recorded every 30 min in the first stage of labour and every 5 min in the second stage of labour to identify fetal distress.

To ensure the quality of data to be gathered from the study subjects, the questionnaire was translated first to the local language and then translated back to English by experts to check its consistency. The instruments were pretested on 5% of the sample size in similar settings, and necessary modifications were made based on the nature of gaps identified in the questionnaire. Additionally, data collectors and supervisors were trained for a day by the investigators on the content of the questionnaire and the ways to collect the data. Moreover, the supervisors and the investigators closely followed the day-to-day data collection process during the pretest and the actual data collection. Furthermore, the filled questionnaire was collected and signed by the supervisor after it was checked for any missing items and completeness. Moreover, cross check was done on 10% of the sample size.

### Measurement

#### Birth asphyxia

Birth asphyxia was considered when the Apgar score was < 7 at the 5th minutes. The trained data collectors (midwives) in the maternity ward and operating theatres performed the scoring and the information was documented on an Apgar score card.

#### Prolonged second stage labor

Prolonged if it exceeds 3 h with provision of regional anaesthesia, or 2 h in the absence of regional anaesthesia for nulliparas. It is multiparous if it exceeds 2 h with regional anaesthesia or 1 h without it. We have assessed this in numbers. Finally, the labor is classified as prolonged or not prolonged based on the definition.

#### Nuchal cord

Nuchal cord was defined as a loop of umbilical cord ≥ 360 degree around the fetal neck. At the time of delivery, each birth was recorded as having a tight nuchal cord, or no tight nuchal cord. ‘Tight nuchal cord’ was defined as the inability to manually reduce the loop over the fetal head.

### Data processing and analysis

The data were entered into EpiData software (version 3.1) and analyzed using SPPS software (version 24.0). Descriptive statistics, frequency, and proportions were computed to summarize the data. Logistic regression analyses were conducted to identify contributing factors to birth asphyxia. Initially, bivariate logistic regression analysis was performed on all independent variables. Multivariable logistic regression was then performed on variables that had a *p*-value ≤ 0.25 in the bivariate logistic regression analysis. The degree of association between independent, and dependent variables were assessed using odds ratio with 95% confidence interval.

## Results

### Socio-demographic characteristics

A total of 279 participants were involved in this study, wherein the response rate was 100%. Approximately, 83.9% of the participating mothers were aged 25–34 years. The majority of the participants were married 271 (97.1%), 224 (80.3%) of them were Hadiya ethnics, 209 (74.9%) were protestants in terms of their religion, and 185 (66.3%) were housewives. Regarding their educational status, 54 (19.4%) of them had pursued higher education, and 122 (43.7%) were rural residents (Table 1).

**Table 1:** Sociodemographic characteristics of mothers in the Nigist Eleni Mohammed Memorial Teaching Hospital, Southern Ethiopia, 2019

Variable		Frequency (*N* = 279)	Percent
Age group	15–19	17	6.1
	20–34	234	83.9
	≥35	28	10.0
Residence	Urban	157	56.3
	Rural	122	43.7
Marital status	Married	271	97.1
	Other	8	2.9
Religion	Protestant	209	74.9
	Orthodox	34	12.2
	Muslim	30	10.8
	Catholic	6	2.2
Ethnicity	Hadiya	224	80.3
	Kambata	28	10.0
	Silte	18	6.5
	other (specify)	9	3.2
Educational status	Able to read and write	29	10.4
	Primary school completed	101	36.2
	High and preparatory school	95	34.1
	Higher institution	54	19.4
Occupation	Government employee	41	14.7
	Merchant	43	15.4
	Housewife	185	66.3
	Others^a^	10	3.6

^a^private, student

### Obstetrics, medical, and fetal characteristics

Among the 279 study participants, 189 (67.7%) of them were multigravida and another 90 (32%) were primigravida. Twenty four (12.5%) participants had ever experienced abortion, while 23 (12.4%) of them had a history of still birth. The majority of the mothers, i.e. 252 (90.3%) participants, were at the gestational age of ≥37 weeks, while 157 (56.3%) had ≥ four antenatal care visits. Furthermore, 44 (15.8%) had faced pregnancy-related complications, wherein 13 (29.5%), 12 (27.3%), 11 (15%), 5 (11.4%), and 3 (6.8%) encountered antepartum hemorrhage, hypertensive disorders, premature rupture of fetal membranes, hyperemesis gravidarum, and other, respectively. In this study, the overall prevalence rate of birth asphyxia was found to be 42(15.1%)**.** Other obstetrics, medical and fetal characteristics are shown in Table 2.

**Table 2:** Obstetrics, medical, and fetal characteristics in the Nigist Eleni Mohammed Memorial Teaching Hospital, Southern Ethiopia, 2019

Variables		Frequency	Percent
Stage of labor at admission (*n* = 271)	First stage	260	96.0
	Second stage	11	4.0
Status of labor (*n* = 279)	Spontaneous	254	91.0
	Induction	17	6.1
	Elective CS	8	2.9
Mode of current delivery	Normal vaginal	224	80.3
	Caesarian section	40	14.3
	Operative vaginal	15	5.4
Augmentation (*n* = 254)	N0	232	91.3
	Yes	22	8.6
Prolonged second stage of labor (*n* = 271)	No	245	90.4
	Yes	26	9.6
Malpresentation/malpresentation (*n* = 271)	No	258	95.2
	Yes	13	4.8
Obstructed labor (*n* = 271)	No	252	93
	Yes	19	7
Sex of newborns (*n* = 279)	Female	130	46.6
	Male	149	53.4
Birth weight of newborn babies (*n* = 279)	Low birth weight (<2500g)	34	12.2
	Normal birth weight (2500-4000 g)	224	80.3
	Macrosomia (>4000g)	21	7.5
Number of delivery/fetuses (*n* = 279)	Single fetus	270	96.8
	Twin	9	3.2
Resuscitation (*n* = 279)	No	247	88.5
	Yes	32	11.5
Fetal Presentation (*n* = 279)	Vertex presentation	264	94.6
	Breech	9	3.2
	Others	6	2.2
Tight nuchal cord (*n* = 279) =	No	250	89.6
	Yes	29	10.4
Meconium stained amniotic fluid (*n* = 279)	Yes	27	9.7
	No	252	90.3
Chronic medical illness (*n* = 279)	No	268	96.1
	Yes	11	3.9
Acute febrile illness during pregnancy (*n* = 279)	No	227	81.4
	Yes	52	18.6
Hemoglobin Level (intrapartum) (*n* = 261)	≥11 g/dl	219	78.5
	<11 g/dl	42	15.1

### Contributing factors of birth asphyxia

The outcome of the bivariate logistic regression analysis showed that, mothers aged ≥35, primigravida, pregnancy related complication, primigravida, prolonged labour, preterm birth, meconium stained amniotic fluid and tight nuchal cord were contributing factors of birth asphyxia. However, in multivariable logistic regression analysis, mothers aged ≥35, primigravida, prolonged second stage of labor, preterm birth, meconium stained amniotic fluid and tight nuchal cord were contributing factors of birth asphyxia. Mothers aged ≥35 were more or six times more likely to have experienced birth asphyxia respect to women in the age group between 20 and 34 years old (AOR = 6.4; 95% CI = 2.0–20.5). Similarly, the prolonged second stage of labor was 4.6 times more likely to have a birth asphyxia than their counterparts (AOR = 4.6; 95%CI =1.6–13.3). Additionally, birth asphyxia was five times more likely to occur in primigravida in comparison to their counterparts (AOR = 5.1; 95% CI =2.0–13.3). Furthermore, preterm birth was nearly 5 times more likely to develop birth asphyxia respect to their counterpart (AOR = 4.7; 95% CI =1.5–14.1). Moreover, meconium stained amniotic fluid were 7.5 times more likely faced births asphyxia than their counterparts (AOR = 7.5; 95% CI =2.5–21.4). Furthermore, tight nuchal cord were more or 3 times more likely to develop birth asphyxia respect to their counterpart (AOR = 3.1; 95% CI =1.2–9.3) (Table 3).

**Table 3:** Contributing factors of birth asphyxia at the Negest Eleni Mohammed Memorial Teaching Hospital, Southern Ethiopia, 2019

Variables		Birth asphyxia		COR (95%CI)	AOR (95%CI)
		No	Yes		
Age group	<20	14	3	1.5 (.4, 5.4)*	.6 (.1, 2.5)
	20–34 (ref.)	204	30	1	1
	≥35	19	9	3.2 (1.3, 7.8)*	6.4 (2.0, 20.5)**
Residence	Urban (ref.)	137	20	1	1
	Rural	100	22	1.5 (.8, 2.9)*	.9 (.4, 2.1)
Gravidity	Primigravida	71	19	1.9 (1.0, 3.8)*	5.1 (2.0, 13.3)**
	Multigravida (ref.)	166	23	1	1
Pregnancy related complications	No (ref.)	205	30	1	1
	Yes	32	12	2.6 (1.2.5.5)*	2.0 (.7, 5.9)
Prolonged second stage of labour	No (ref.)	216	29	1	1
	Yes	16	10	4.7 (2.0, 11.2)*	4.6 (1.6, 13.3)**
Gestational age	≥37 weeks (ref.)	219	33	1	1
	<37 weeks	18	9	3.3 (1.4, 8.0)*	4.7 (1.5,14.1)**
Color of amniotic fluid	Clear (ref.)	222	30	1	1
	Meconium stained	15	12	5.9 (2.5, 13.8)*	7.5 (2.5, 21.4)**
Tight nuchal cord	No (ref.)	218	32	1	1
	Yes	19	10	3.6 (1.5,8.4)*	3.1 (1.2, 9.3)**

## Discussion

In this study, the overall prevalence of birth asphyxia was found to be 15.1%. This is higher than seen in studies conducted in Jimma, Dire Dawa, and Tanzania which were12.5, 3.6, and 11.5%, respectively [15, 33, 34]. The increased prevalence may be due to the hospitals from the study being referral hospitals, which had inferior health facilities. Further, this difference could be an indicator of the ineffectiveness of national strategies for maternal and newborn care services. In contrast, this study found a lower prevalence of birth asphyxia as compared to studies carried out in Zambia and Nigeria which were 23 and 24.7%, respectively [8, 35]. The variation in our study might be due to differences in study design, setting, sample size and accessible maternal health care services.

In this study, mothers aged ≥ 35 were one of the contributing factors to birth asphyxia rates. The following discovery was very nearly found to be a universal fact: the increased age of the mother increased the risk of several adverse outcomes [24, 36, 37]. Similar findings were also reported from a study carried out in Cameroon [20] which revealed that mothers aged ≥ 35 were more likely to experience a birth asphyxia. However, in contrast to what was found in Ethiopia [19], and Pakistan [13, 22], a younger maternal age was not found to be a contributing factor of birth asphyxia in this study.

Primigravida was found to be a contributing factor of birth asphyxia and this was consistent with the research conducted in India [14], Ethiopia [15], and Pakistan [18, 22]. The most common obstetric complication was failure to progress in labor [21], which may subsequently result in the delivery of an asphyxiated baby.

This study also showed that mothers encountering a prolonged second stage of labor were at a greater risk for birth asphyxia with respect to their counterparts. These findings were similar to those of studies conducted in Ethiopia, Colombia and Pakistan [15, 16, 22]. One reason that babies born after a prolonged labor have a greater risk of birth asphyxia may be due to umbilical cord problems or the stress of too many contractions.

Meconium stained amniotic fluid was a contributing factor to birth asphyxia, similar to what was reported in Ethiopia [15], India [17], and Pakistan [18]. Possibly, the presence of meconium in the amniotic fluid may cause aspiration of meconium-stained amniotic fluid to occur, and this can block small airways, deactivate surfactants and may also inhibit surfactant synthesis, resulting in birth asphyxia [38, 39]. As shown by the present study, prematurity was found to have a significant association with birth asphyxia, which is in line with what has been found in Ethiopia [15] and Pakistan [18, 22]. A baby being born before 37 completed weeks of pregnancy may develop birth asphyxia due to an insufficient amount of surfactant in the lungs. Surfactant creates a continuously reforming surface layer over the alveoli which reduces surface tension, prevents atelectasis and maintains alveolar stability. A deficiency in pulmonary surfactant production causes Respiratory Distress Syndrome [40, 41].

Birth asphyxia was also found to be associated with a tight nuchal cord. This finding is similar to that of the studies conducted in Ethiopia [15] and Pakistan [22]. This might be related to tight nuchal cord cords can interrupt normal blood, nutrient, and oxygen exchange by compressing the umbilical cord, or restricts arteries and veins in the fetal neck, this can lead to birth asphyxia [42, 43].

## Limitations of the study

In this study, the Apgar score was used to diagnose asphyxia. However, using the Apgar score alone to diagnose birth asphyxia has been found to be inappropriate as it is an expression of the infant’s physiological condition at one point in time, which includes subjective components. Also, the study relied on hospital deliveries. However, several deliveries occurred outside the hospital, making it difficult to capture data such as birth asphyxia, so the estimates contained in this study may have underreported the cases at the community level. Additionally, sometimes women who came to the hospital may encounter labor complications, compared to women that deliver out of hospital. Therefore, this may also cause overestimation of the prevalence of birth asphyxia.

## Conclusion

Birth asphyxia is still prevalent in the study setting. Primigravida, prolonged labour, preterm birth, and mothers aged ≥35 meconium stained amniotic fluid and tight nuchal cord were contributing factors of birth asphyxia. Results of this study show the need for the better maternal care, creating awareness about contributing factors of birth asphyxia to the maternity health professionals, careful monitoring of labor, identifying and taking proper measure could help in decreasing the occurrence of birth asphyxia. The results of the study will help policy-makers, program designers and Non-governmental organizations to support the study area. Additionally, the findings of the study can also help as a secondary data for further study in same area of inquiry.

## Data Availability

The datasets used and /or analysed during the current study are available from the corresponding author on reasonable request.

## Abbreviations

AOR: Adjusted odd ratio
CI: Confidence interval
COR: crude odds ratio
FMOH: Federal ministry of health
PMTCT: Prevention of mother-to-child transmission of HIV
SPSS: statistical package for social sciences
WHO: World Health Organisation
